# Crosstalk between hydroxytyrosol, a major olive oil phenol, and HIF-1 in MCF-7 breast cancer cells

**DOI:** 10.1038/s41598-020-63417-6

**Published:** 2020-04-14

**Authors:** Jesús Calahorra, Esther Martínez-Lara, José M. Granadino-Roldán, Juan M. Martí, Ana Cañuelo, Santos Blanco, F. Javier Oliver, Eva Siles

**Affiliations:** 1 Departamento de Biología Experimental, Universidad de Jaén, Campus Las Lagunillas s/n, Jaén, 23071 Spain; 2 Departamento de Química Física y Analítica, Universidad de Jaén, Campus Las Lagunillas s/n, Jaén, 23071 Spain; 3 Instituto López Neyra de Parasitología y Biomedicina, IPBLN, CSIC PTS-Granada, Armilla, 18016 Spain

**Keywords:** Breast cancer, Breast cancer

## Abstract

Olive oil intake has been linked with a lower incidence of breast cancer. Hypoxic microenvironment in solid tumors, such as breast cancer, is known to play a crucial role in cancer progression and in the failure of anticancer treatments. HIF-1 is the foremost effector in hypoxic response, and given that hydroxytyrosol (HT) is one of the main bioactive compounds in olive oil, in this study we deepen into its modulatory role on HIF-1. Our results in MCF-7 breast cancer cells demonstrate that HT decreases HIF-1α protein, probably by downregulating oxidative stress and by inhibiting the PI3K/Akt/mTOR pathway. Strikingly, the expression of HIF-1 target genes does not show a parallel decrease. Particularly, adrenomedullin and vascular endothelial growth factor are up-regulated by high concentrations of HT even in HIF-1α silenced cells, pointing to HIF-1-independent mechanisms of regulation. In fact, we show, by i*n silico* modelling and transcriptional analysis, that high doses of HT may act as an agonist of the aryl hydrocarbon receptor favoring the induction of these angiogenic genes. In conclusion, we suggest that the effect of HT in a hypoxic environment is largely affected by its concentration and involves both HIF-1 dependent and independent mechanisms.

## Introduction

Breast cancer is the most common cancer diagnosed in women and the second leading cause of death from cancer among them^1^. Approximately 25%–40% of invasive breast cancers exhibit hypoxic regions in which oxygen pressure is diminished^2^. This hypoxic microenvironment favors cancer progression and metastasis and should be taken into account in cancer research.

Hypoxia inducible factor-1 (HIF-1) is the key factor in the adaptive response to hypoxia. The binding of HIF-1 to hypoxic response elements (HREs) regulates the transcription of a plethora of genes involved, among others, in glucose metabolism, cell proliferation, cell survival and angiogenesis^3^. Therefore, HIF-1 overexpression is strongly related with poor prognosis and resistance to chemotherapy and radiotherapy^4,5^. HIF-1 is a heterodimeric transcription factor composed by an oxygen-sensitive α subunit, HIF-1α, and a constitutive subunit, ARNT. HIF-1α transcription, translation and stability are highly regulated in an oxygen-dependent and independent manner. At the transcriptional level, HIF-1α expression is regulated by different transcription factors such as Sp1, NF-κB, Erg1 and by HIF-1 itself^6–8^. The translation of HIF-1α mRNA can be upregulated through the PI3K/Akt/mTOR pathway. Particularly, mTOR when activated by Akt, mediates the phosphorylation of p70 S6 kinase (S6K) that induces HIF-1α translation through the ribosomal protein S6^9^. Once translated, and under normoxic conditions, HIF-1α subunit is quickly degraded due to the activity of the prolyl-4-hydroxylases (PHDs) that label HIF-1α enabling its ubiquitination by the pVHL and its degradation in the proteasome. Conversely, hypoxic conditions inhibit PHDs activity and promote HIF-1α stabilization. Besides this oxygen-dependent regulation of HIF-1α, reactive oxygen species (ROS) and nitric oxide (NO), crucial in tumorigenesis and particularly high in cancer cells, also contribute to the stabilization of HIF-1α by inhibiting PHDs activity under both hypoxic and normoxic conditions^10,11^. Finally, the transcriptional activity of HIF-1 can be modulated by the factor inhibiting HIF-1 (FIH) which hydroxylates a critical asparagine residue (Asn-803), in an oxygen-dependent manner, blocking coactivator recruitment^12^. HIF-1α is also dependent on the expression and activity of Poly(ADP-ribose) polymerase-1 (PARP-1)^13,14^, a nuclear, zinc-finger, DNA-binding protein activated in response to oxidative and nitrosative stress. This enzyme plays an important role in a number of processes such as DNA repair, chromatin remodeling, transcription or regulation of the cell cycle, among others^15^. PARP inhibition has been demonstrated to exert beneficial effects hindering prometastasic activities and adaptation of tumor to different microenvironments such as hypoxia^16^.

Lifestyle factors play a significant role in the risk of suffering breast cancer^17^. The PREDIMED study, a large dietary intervention trial, showed the beneficial effect of a Mediterranean diet supplemented with extra-virgin olive oil (EVOO) in the primary prevention of breast cancer^18^. Olive oil is mainly composed of fatty acids, particularly oleic acid, but its mechanical extraction at temperatures lower than 30 °C also afford a high concentration of different minor components, such as phenols. The main phenolic alcohol is hydroxytyrosol (HT). Several studies have demonstrated the beneficial properties of this compound in a number of models and cancer linked events^19^, particularly in breast cancer^20–22^. In fact, we have recently described that hypoxia modulates the antioxidant effect of HT in MCF-7 breast cancer cells^23^. With this background, and considering the importance of hypoxia and HIF-1 in breast cancer progression and response to anticancer treatments, the aim of the present study is to investigate the effect of HT in the expression and transcriptional activity of this protein. Our results indicate that in hypoxic MCF-7 breast cancer cells, HT decreases the expression of HIF-1α, an effect probably linked to its antioxidant action and to the down-regulation of the PI3K/Akt/mTOR pathway. Moreover, we show that at high concentrations HT can even act as an AHR agonist.

## Results

### Effect of HT on hypoxic MCF-7 cell viability

**Figure 1:**
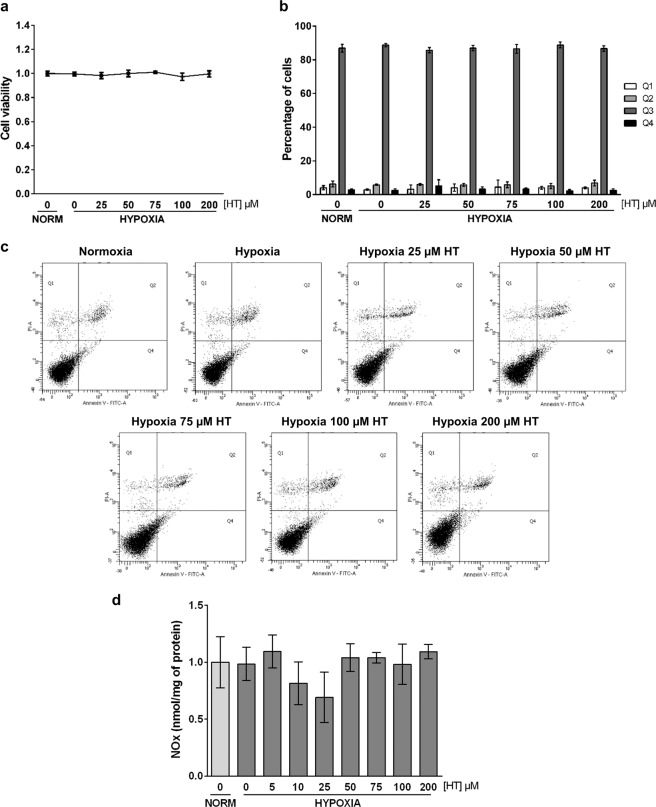
HT does not affect cell viability or nitric oxide levels in hypoxic conditions. (a) Cell viability measured as ratio of living cells (trypan blue unstained cells) relative to normoxic non HT-treated cells. (b) Representative dot plots of Annexin V/PI FACS analysis: Q1, necrotic cells (Annexin V negative and PI positive); Q2, late apoptotic cells (Annexin V and PI positive); Q3, viable cells (Annexin V and PI negative cells) and Q4, early apoptotic cells (Annexin V positive and PI negative cells). (c) NO levels (nmol/mg of protein) relative to normoxic HT-untreated cells. Values represent the mean ± SD from three independent experiments.

We previously reported^23^, by using the sulforhodamine B assay, that the treatment of hypoxic MCF-7 cells with concentrations of HT up to 400 µM did not affect cell proliferation. In order to further corroborate the absence of toxicity of the HT concentrations used in this study (0–200 µM), we performed trypan blue exclusion (cell viability, Fig. 1a) and Annexin-V/IP FACS (apoptosis, Fig. 1b) assays. The results revealed no significant changes in viable nor apoptotic or necrotic cells, confirming the absence of a toxic effect of HT in our experimental conditions.

### HT does not affect nitric oxide levels during hypoxia

We have described^23^ that a sub-cytotoxic treatment of hypoxic MCF-7 cells with HT (5–200 μM, 16 h) decreased the oxidative stress level. NO is also crucial in the response to hypoxia. Therefore, we have evaluated the effect of those same concentrations of HT in NO production. Conversely to what we previously described for ROS, HT did not affect NO levels in a significant manner (Fig. 1c).

### PARP-1 activity is decreased by high doses of HT

**Figure 2:**
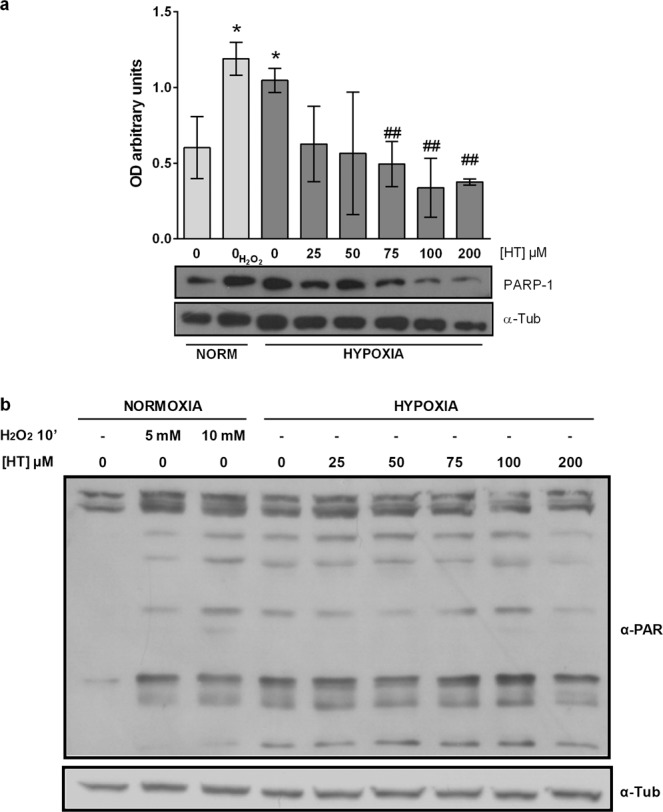
HT decreases PARP-1 protein level and activity. (**a**) Densitometric quantifications of PARP-1, protein level relative to α-tubulin (α-Tub). A representative immunoblot is shown. Values represent the mean ± SD from three independent experiments. Statistically significant differences with the corresponding non-treated normoxic cells: *p < 0.05. Statistically significant differences with the corresponding non-treated hypoxic cells: ^##^p < 0.01. (**b**) α-PAR representative immunoblot.

ROS and NO damage DNA and modulate the activity of PARP-1, a protein largely involved in cancer progression that also regulates HIF-1α response. To assess whether the antioxidant effect of HT treatment decreased the expression and activity of this enzyme, we evaluated PARP-1 levels and PARylated proteins by using specific antibodies. As shown in Fig. 2, the expression of PARP-1 was increased in hypoxic conditions but returned to basal levels when cells were treated with concentrations of HT equal to or greater than 75 µM. Similarly, PARylated proteins in hypoxic cells were also decreased by HT but only at 200 µM.

### HT reduces HIF-1*α* stability in a dose dependent manner, in part, through the mTOR pathway

**Figure 3:**
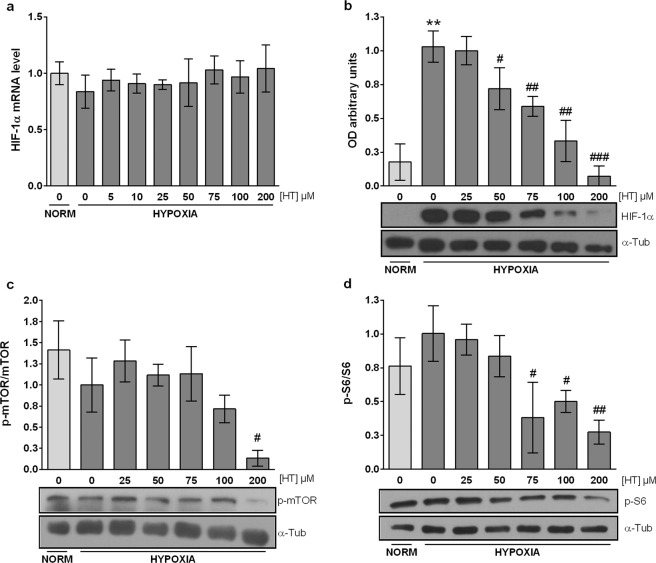
HT down-regulates HIF-1α in a dose dependent manner: m-TOR pathway involvement (**a**) Effect of HT on HIF-1α mRNA levels relative to hypoxic non HT-treated cells after normalization against PPIA. (**b**) Densitometric quantifications of HIF-1α relative to α-tubulin protein level (α-Tub). Densitometric quantifications of p-mTOR (**c**) and p-S6 (**d**) relative to unphosphorylated corresponding proteins (Supplementary Fig. 1). A representative immunoblot is shown. Values represent the mean ± SD from three independent experiments. Statistically significant differences with the corresponding non-treated normoxic cells: **p < 0.01. Statistically significant differences with the corresponding non-treated hypoxic cells: ^#^p < 0.05, ^##^p < 0.01, ^###^p < 0.001.

Although no changes were detected in NO levels (Fig. 1c), the impact of HT treatment on both oxidative stress and PARP-1 led us to evaluate the mRNA and protein level of HIF-1α. No effects were detected on the expression of HIF-1α mRNA, suggesting that HT does not modulate the transcription of this gene (Fig. 3a). However, the western-blot analysis revealed that HT was able to reduce HIF-1α protein levels in a dose dependent manner from 50 µM to 200 µM (Fig. 3b). In order to further investigate the mechanism underlying this effect, we analyzed the impact of HT on the activation of the mTOR pathway. The active form of mTOR (p-mTOR) was decreased by treatment with HT 200 µM (Fig. 3c and Supplementary Fig. 1a). Its downstream activated target p-S6 (Fig. 3d and Supplementary Fig. 1b) was reduced even at lower concentrations (HT 75, 100 and 200 µM).

### HIF-1 targets are up-regulated by high concentrations of HT

**Figure 4:**
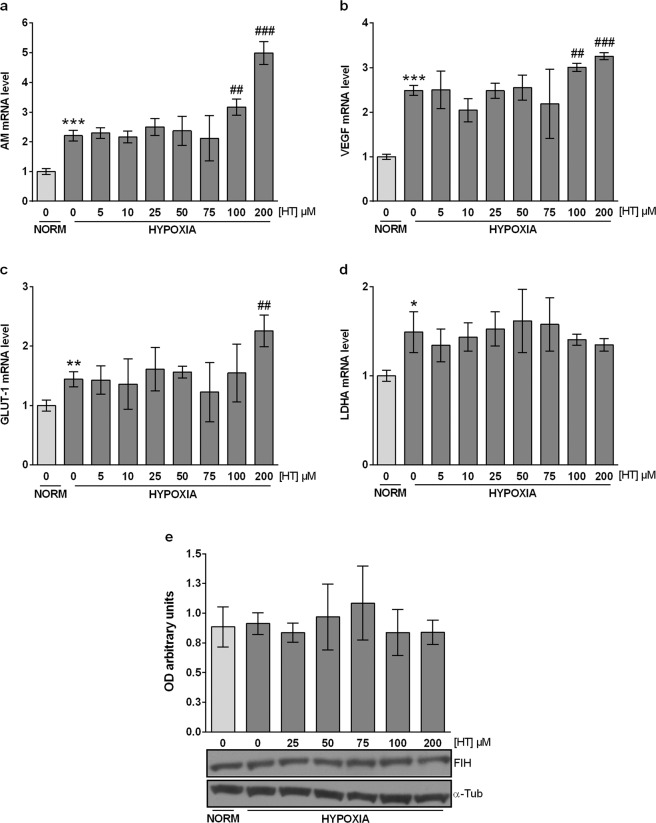
The effect of HT on HIF-1 targets does not parallel HIF-1α expression. AM (**a**), VEGF (**b**), GLUT-1 (**c**) and (**d**) LDHA mRNA levels. Results are expressed as mRNA expression relative to normoxic non HT-treated cells after normalization against PPIA. (**e**) The up-regulation of HIF-1α targets by HT is not due to FIH inhibition. Densitometric quantifications of FIH protein level relative to α-tubulin (α-Tub). A representative immunoblot is shown. Values represent the mean ± SD from three independent experiments. Statistically significant differences with the corresponding non-treated normoxic cells: *p < 0.05, **p < 0.01, ***p < 0.001. Statistically significant differences with the corresponding non-treated hypoxic cells: ^##^p < 0.01, ^###^p < 0.001.

We next evaluated the effect of HT on the transcriptional activity of HIF-1. For that purpose, we analyzed the mRNA levels of the angiogenic targets adrenomedullin (AM) and vascular endothelial growth factor (VEGF), and of the metabolic targets glucose transporter-1 (GLUT-1) and lactate dehydrogenase A (LDHA). As expected, the expression of all these genes was up-regulated under hypoxia (Fig. 4). Surprisingly, and despite HIF-1α protein was down-regulated by HT treatment, the two highest concentration of this phenol (100 and 200 µM) promoted the up-regulation of AM, VEGF and GLUT-1. Hence, the transcriptional activity of HIF-1 and the protein levels of HIF-1α do not follow a similar pattern of response when MCF-7 cells are treated with high concentrations of HT.

### The up-regulation of HIF-1α targets by HT is not due to FIH inhibition

The transcriptional activity of HIF-1 is modulated by FIH. The opposite effect of HT in the expression and transcriptional activity of HIF-1 led us to evaluate the influence of this phenol on FIH (Fig. 4e). No changes in the expression of this protein were observed suggesting that the up-regulation of AM, VEGF and GLUT-1 cannot be attributed to a lower expression of FIH.

### GLUT-1 but not AM and VEGF overexpression by HT, is HIF-1α dependent

**Figure 5:**
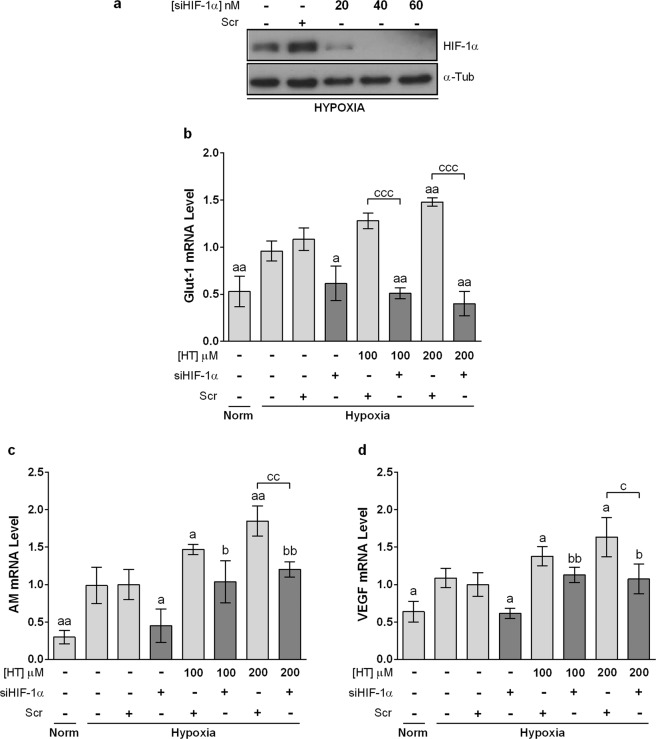
HIF-1α silencing abolishes HT-dependent GLUT-1 upregulation but not AM and VEGF induction. (**a**) Representative immunoblot of HIF-1α knockdown with different concentrations of siHIF-1α. Effect of HIF-1α –silencing (siHIF-1α 40 nM) on the transcription level of (**b**) GLUT-1, (**c**) AM and (**d**) VEGF in hypoxic cells after treatment with HT 100 and 200 μM. Results are expressed as mRNA levels relative to Scr hypoxic cells after normalization against PPIA. Values represent the mean ± SD from three independent experiments. Statistically significant differences with the corresponding Scr-siRNA transfected hypoxic cells: ^a^p < 0.05, ^aa^p < 0.01. Statistically significant differences with the corresponding HIF-1α-silenced cells: ^b^p < 0.05, ^bb^p < 0.01. Statistically significant differences with the corresponding HT treated-non-silenced cells: ^c^p < 0.05, ^cc^p < 0.01, ^ccc^p < 0.001.

AM, VEGF and GLUT-1 have been consistently described as HIF-1 target genes. However, the HIF-1 pathway did not seem to explain their overexpression after treatment with HT 100 and 200 µM. In order to determine the implication of HIF-1 in such overexpression we analyzed whether the up-regulation of AM, VEGF and GLUT-1 persisted after knocking down HIF-1α (Fig. 5a). As shown in Fig. 5b, the silencing of HIF-1α abrogated the HT-induced overexpression of GLUT-1. However, AM and VEGF genes remained overexpressed in HT-treated cells after silencing HIF-1α (Fig. 5c, d). These results indicate that HT exerts its transcriptional regulation of AM, VEGF through HIF-1-dependent and independent mechanisms.

### HIF-1-independent pathways also mediate the effect of HT on the hypoxic response

**Figure 6:**
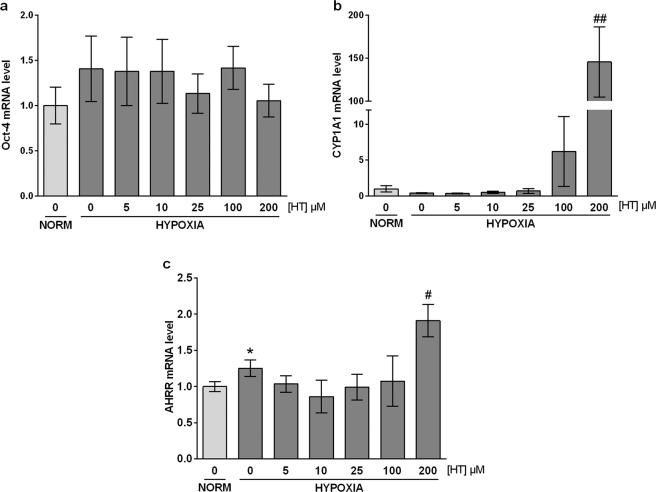
HT does not modulate HIF-2 but activates AHR at high concentrations. mRNA levels of Oct-4 (**a**), CYP1A1 (**b**) and AHRR (**c**). Results are expressed as mRNA expression relative to normoxic non HT-treated cells after normalization against PPIA. Values represent the mean ± SD from three independent experiments. Statistically significant differences with the corresponding non-treated normoxic cells: *p < 0.05. Statistically significant differences with the corresponding non-treated hypoxic cells: ^#^p < 0.05, ^##^p < 0.01.

**Figure 7:**
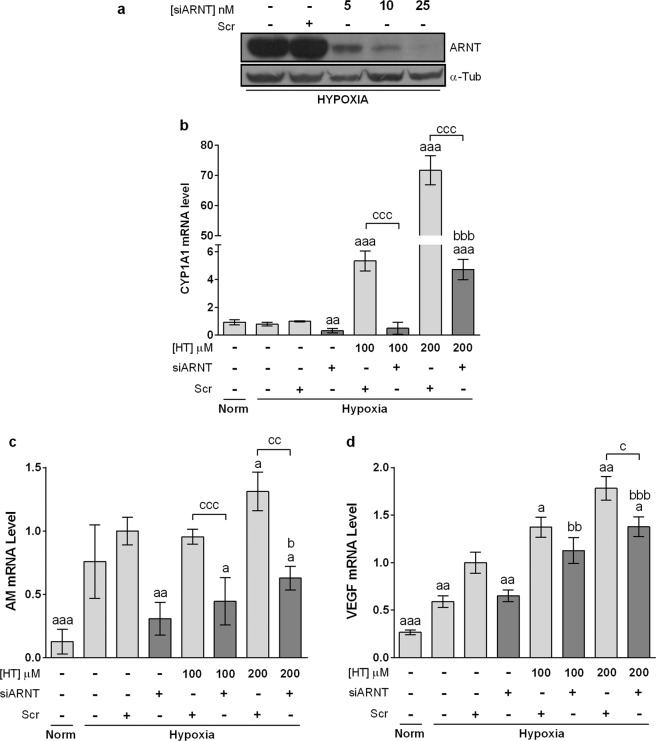
ARNT seems to be involved in the effect of HT. (**a**) Immunoblot of ARNT knockdown with siARNT. Effect of ARNT silencing on the transcription level of (**b**) CYP1A1, (**c**) AM and (**d**) VEGF in hypoxic cells after treatment with HT 100 and 200 μM. Results are expressed as mRNA levels relative to Scr hipoxic cells after normalization against PPIA. Values represent the mean ± SD from three independent experiments. Statistically significant differences with the corresponding Scr hypoxic cells: ^a^p < 0.05, ^aa^p < 0.01, ^aaa^p < 0.001. Statistically significant differences with the corresponding ARNT-silenced cells: ^b^p < 0.05, ^bb^p < 0.01, ^bbb^p < 0.001. Statistically significant differences with the corresponding HT treated-non-silenced cells: ^c^p < 0.05, ^cc^p < 0.01, ^ccc^p < 0.001.

Apart from HIF-1, HIF-2 is also involved in the up-regulation of certain genes in response to a hypoxic stimulus. Thus, we next addressed the possible involvement of HIF-2 in the transcriptional response to HT. For that purpose, we quantified the expression of the specific HIF-2 target Oct-4 in hypoxic HT-treated cells. As shown in Fig. 6a, HT produced no effect on the mRNA level of this gene, suggesting that HIF-2α is not involved in the up-regulation of AM and VEGF. It has been previously reported that AM and VEGF genes contain xenobiotic response elements (XRE)^24,25^ and therefore can be regulated by the aryl hydrocarbon receptor (AHR). CYP1A1 is a sensitive marker of AHR activation and we found that it was intensely overexpressed at high HT doses (Fig. 6b). The AHR repressor (AHRR) is also known to be induced in response to AHR activation^26^ and, in agreement with the previous result, its expression was also induced at high HT concentrations (Fig. 6c). These results seem to indicate that at high concentrations HT could act as an AHR ligand, inducing AM and VEGF expression. AHR and HIF-1α must heterodimerize with ARNT to carry out its transcriptional activity. Therefore, by silencing ARNT the effect of both HIF-1 and AHR would be concomitantly abolished. As shown in Fig. 7b, the effect of HT on CYP1A1 was almost completely abolished in ARNT-silenced hypoxic cells, further corroborating that at high concentrations HT binds and activates the AHR pathway. However, the effect of HT on AM or VEGF although significantly decreased was not completely abrogated (Figs. 7c and 7d), suggesting the involvement of additional mechanisms of regulation, not linked with ARNT.

### *In silico* modelling of HT interaction with AHR

**Figure 8:**
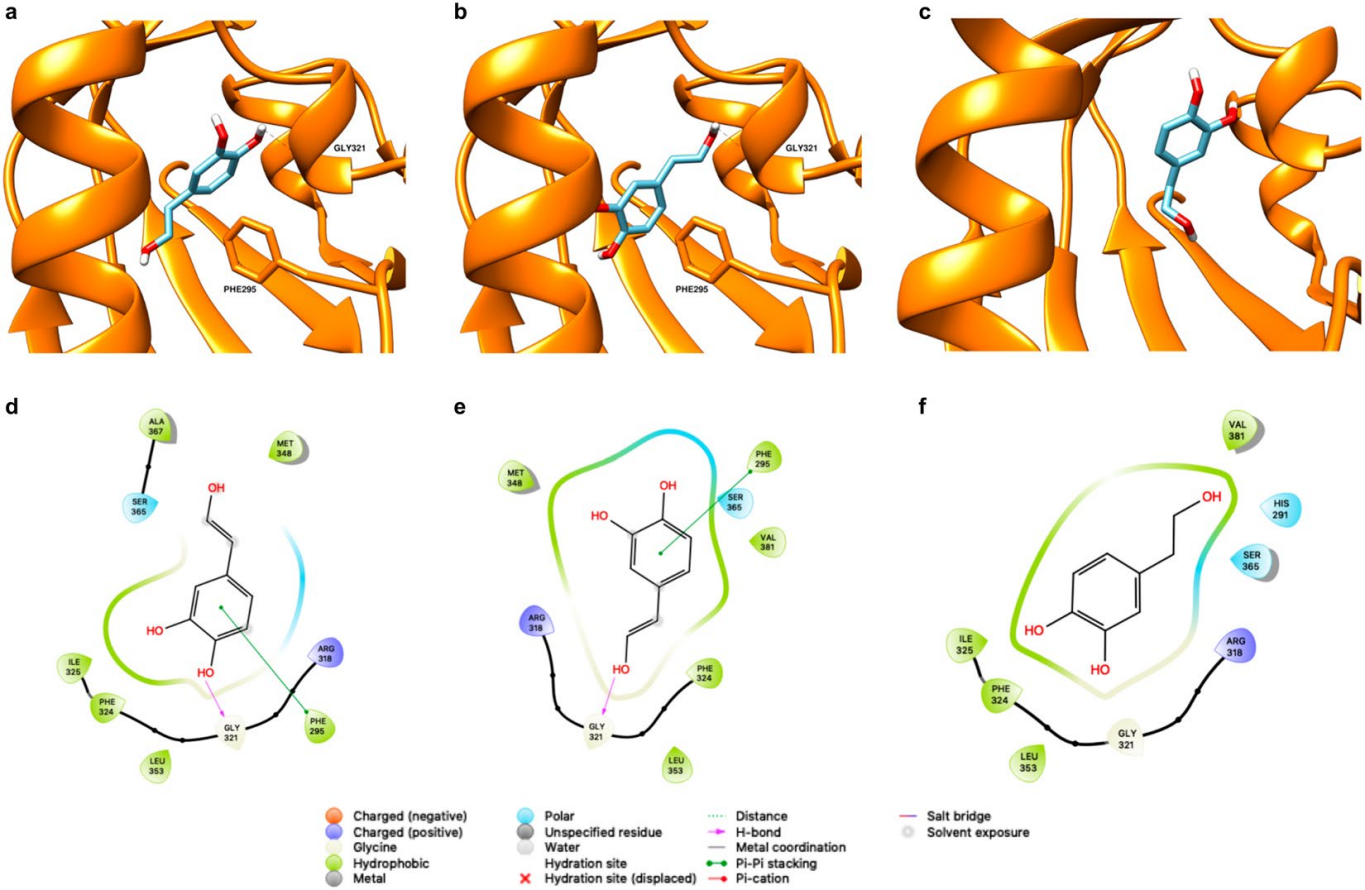
HT docked into human AHR as predicted by Autodock Vina (**a,b**) or BindScope (**c**), hydrogens other than those of the OH groups of HT are omitted for clarity. (**d–f**) 2D representations of the corresponding docked poses highlighting the interactions established between the ligand and the protein.

The large central pocket of the AHR PAS-B domain has been shown to promiscuously bind a number of toxic halogenated aromatic hydrocarbons, polycyclic aromatic hydrocarbons, and other natural, endogenous or synthetic agonists and antagonists^27–29^, being 2,3,7,8-tetrachlorodibenzo-p-dioxin (TCDD) the most potent ligand. In order to support our results pointing that HT is an AHR ligand, we performed a docking analysis. As it has been shown that there exist important differences between the *apo* and *holo* structures of the HIF-2α PAS-B domain, used as template for homology modelling of the AHR PAS-B domain^30,31^, we obtained a model for our docking analysis using three *holo* structures of the HIF-2α domain^32^. We have used and compared two different docking methodologies, one rigid docking that uses a pre-defined binding cavity and the Autodock Vina program^33^, and another one that does not need the binding pocket to be defined, based on deep neural networks, using the web program Bindscope^34^. The first methodology rendered ten possible poses that were scored both with the Autodock Vina scoring function and with 3D-convolutional neural networks, using the web program K_DEEP_^35^. The two best-scored consensus poses are shown, together with the pose obtained by Bindscope, in Fig. 8. Interestingly, Bindscope, which is not biased by a pre-defined binding site, predicted HT to interact with the same parts of the protein of our defined binding site, although this pose reflects a different orientation as compared to those obtained with Autodock Vina, which showed two poses in which the main difference is which OH group is establishing a hydrogen bond with GLY321. In all cases, the poses predicted for HT establish interactions with residues which have been proven to form the so-called TCDD binding fingerprint^36^ or others proved to be important for binding^37,38^ (3,4 and 5 of these residues interacting with HT for the two poses from Autodock Vina and the pose from Bindscope, respectively). The poses obtained with Autodock Vina agreed in exhibiting a previously described crucial π-π interaction between HT and PHE295^36^, also obtained in previous docking experiments^39^. Overall these docking results support the findings of HT as ligand of human AHR.

## Discussion

The Mediterranean diet has been linked to a lower incidence of different types of cancer, and particularly of breast cancer^18,20,22,40^. In a previous study we demonstrated that HT, the main EVOO phenolic compound in olive oil and a recognized nutraceutical, modulates the oxidative response to hypoxia of MCF-7 cells^23^, a breast cancer cell line consistently used in the literature to assess the effect of this phenol. In the present study, and considering the crucial role of HIF-1 in hypoxia and in cancer prognosis, we have deepened into the modulatory effect that this phenol exerts in the response of the HIF-1 pathway.

NO and ROS up-regulates HIF-1α levels by impairing PHDs and its pVHL-mediated degradation^11,41^. The effect of HT on the production of NO has been previously reported in a number of studies. Although some of them show that HT results ineffective in reducing NO production^42^, most of them demonstrate that this phenol down-regulates NO levels, and point to the inhibition of the iNOS isoform as the plausible mechanism underlying this effect^43^^.^ However, these studies were carried out in cells grown in normoxia and the data about the effect that HT exerts in NO production in hypoxic cells are scarce. We reported^44^ that in hypoxic non tumor cells NO levels were decreased by treatment with this phenol (100 and 200 µM). Breast cancer cells exhibit NO levels that are particularly high, and decreasing those level would help to counteract several malignancy-related effects including angiogenesis, apoptosis, cell cycle, invasion, and metastasis^45^. In this study we show that HT is unable to exert any effect on NO levels, suggesting that the plausible inhibition of iNOS is overwhelmed in hypoxic MCF-7 cells.

PARP-1 is the funding and most studied member of a family of proteins which catalyzes the synthesis and transfer of negatively charged ADP-ribose moieties to a number of target protein substrates that result PARylated. PARP-1 activity is induced by oxidative and nitrosative stress and the crucial role of this protein in the response to hypoxia, both in non-tumor and tumor cells, has been extensively described by our group^14,46,47^. Particularly, we have reported that the inhibition of PARP-1 decreases the response of HIF-1α. Pharmacological inhibition of PARP-1 provides protection from oxidative stress-associated tissue injury, down-regulates the inflammatory response and is also beneficial in cancer treatment by mechanisms such as selective killing of homologous recombination-deficient tumor cells, down-regulation of tumor-related gene expression (e.g. AP-1 and NF-κB-mediated transcription) and apoptotic threshold in the co-treatment with chemo and radiotherapy^48^. In fact, it is currently being used to treat some breast cancers. There is strong evidence in support of the concept that hypoxia increases both the expression and the activity of PARP-1, contributing to tumor malignancy. Hence, the down-regulation of PARP-1 by HT would be potentially beneficial in cancer patients, e.g. it could counteract the cardiovascular and musculoskeletal complications associated to anti-cancer therapies. Our results corroborate the up-regulation of PARP-1 expression and activity in hypoxic MCF-7 cells, and demonstrate that HT treatment decreases both. However, these results although positive, cannot be exclusively linked to the antioxidant effect of HT, as they are achieved at concentrations (≥75 µM) clearly over the antioxidant ones (≥5 µM)^23^. PARP-1 is also regulated by a number of microRNAs^49,50^, and HT is known to modulate the expression of these small non-coding RNA molecules^51^. Thus, this mechanism could be a plausible additional contributor to PARP-1 down-regulation. Strikingly, PARylated proteins did not decrease in the same manner as PARP-1. This unparallel response could be due to the activity of other PARP proteins, such as PARP-2, also involved in DNA repair and gene transcription.

Breast cancer patients exhibit significantly high HIF-1α levels, which correlate with more aggressive cancer features, and particularly with a poor disease free and overall survival^52^. Our results showed that HT (50–200 µM) decreased HIF-1α protein level without modulating its mRNA expression. These data resemble those previously observed by our group in hypoxic non-tumor renal cells^44^ and by others in human colon adenocarcinoma HT-29 cells grown *in vitro* and *in vivo* in a xenograft model^53^, however the *in vitro* results were obtained after treatment with much higher concentrations of this phenol (400–800 µM)^54^. Although HT induced no changes in NO levels in MCF-7 hypoxic cells, the down-regulation of HIF-1α by HT treatment can be attributed to the antioxidant effect of HT, which reactivates PHD activity. As mentioned above, PARP-1 protein and enzymatic activity have also been linked to lower HIF-1 levels^14,47^. In a skin carcinogenesis model, HIF-1α was one of the genes decreased after pharmacological inhibition of PARP-1^13^. Although the particular mechanism underlying such effect has not been completely elucidated, the inhibition of inflammatory processes, and particularly of NF-κB, has been proposed to play a critical role. The PI3K/Akt/mTOR pathway, an upstream inducer of NFκB, can also regulate HIF-1α expression. Besides, mTOR activity is also modulated by PARP-1. Particularly, upon PARP-1 activation, AMPKα results PARylated and is exported to cytosol where it is phosphorylated by LKB1, a kinase presumably modified in a PARylation-dependent manner as well. Finally, the phosphorylated AMPKα inhibits mTOR^55^. According to the literature, the effect of HT on these pathways is controversial. Some authors have pointed that HT upregulates AMPK^56^ and inhibits Akt phosphorylation in tumor and non-tumor cells at 50, 100 and 200 µM^57–59^. However, HT has also been shown to promote Akt phosphorylation^60^. The data presented here support the hypothesis that high concentrations of HT, shown to decrease PARylation, inhibit the activity of this pathway as lower levels of p-mTOR and p-S6 were observed in hypoxic 200 µM HT-treated cells. Altogether, these results suggest that the down-regulation of HIF-1α by HT is not only achieved post-translationally, through ROS decrease, but also at a translational level through the inhibition of the PI3K/Akt/mTOR pathway. This modulatory effect of HT could contribute to counteract breast cancer progression as high levels of PI3K, pAkt and p-mTOR are predictors of an adverse outcome in breast cancer patients^61,62^. Besides, autophagy, a process of cellular self-digestion, is inhibited by mTOR activation and AMPK inhibition. The predictive value of autophagy for breast cancer prognosis remains unclear. However, inactivation of autophagy has been associated with shortened survival of breast cancer patients^63^ and the loss of autophagy-related genes increases the aggressive development of HER2-positive breast cancer^64^. Several reports point to HT as an inducer of autophagy^65,66^. Although the study of the effect of HT on autophagy in hypoxic conditions is beyond of this work, our results seem to indicate that HT could affect this process. Further studies would be necessary in order to demonstrate this effect.

HIF-1 plays a central role in the adaptive response to hypoxia. It therefore appears crucial to investigate the effect of HT in the expression of some HIF-1 target genes. The induction of angiogenesis and an increased glucose uptake are two key molecular pathways that favor cell survival in a hypoxic environment. Those pathways are induced, among others, by the angiogenic factors VEGF and AM and by the metabolic proteins GLUT-1 and LDH. As shown above, the treatment of MCF-7 cells with HT decreases the expression of HIF-1 α. Thus, we expected those genes to follow a similar pattern of response and to be down-regulated in HT-treated hypoxic cells. Our findings showed that HT exerts no effect on the mRNA levels of LDHA whereas, strikingly, high doses of this phenol up-regulates the transcription of AM, VEGF and GLUT-1, suggesting that HT does not reduce but induces the transcriptional activity of HIF-1. While this result does not match previous studies showing lower levels of VEGF in different models^53,67,68^, it supports our previous findings in renal cells^44^. FIH, through its ability to hydroxylate HIF-1α Asn-803, depresses HIF-1 transcriptional activity^3^. Thus, a plausible decrease in this protein could be responsible for the higher transcriptional activity of HIF-1. However, as the expression of FIH does not seem to be modulated by HT, we suggested that other regulatory pathways, different to HIF-1, are probably underlying the up-regulation of these genes. In order to corroborate the particular involvement of HIF-1 in the response of AM, VEGF and GLUT-1 to HT treatment, we silenced HIF-1α expression by siRNA. The induction of GLUT-1 by HT was completely abolished in these cells, suggesting that its up-regulation after HT treatment is highly dependent on HIF-1 transcriptional activity. However, AM and VEGF were only partially down-regulated, corroborating the involvement of other regulatory mechanisms, additional to HIF-1, in the HT-mediated induction of those genes. HIF-2 is another member of the HIF family also involved in the response to hypoxia. The effect of HT in the activity of this transcription factor is largely unknown. Hence, we wonder whether the reported increase in AM and VEGF could be mediated by HIF-2^69^. To test this hypothesis, we assessed the impact of HT in the transcriptional activity of HIF-2. Erythropoietin or angiopoietin 2 are canonical HIF-2 targets but they are very poorly expressed in MCF-7 cells^70^, therefore we evaluated the mRNA levels of another specific HIF-2α target, Oct-4^71^. HT did not affect Oct-4 transcription so HIF-2 does not seem to be involved in the response of AM and VEGF to HT. AM and VEGF can also be up-regulated by the hydrocarbon receptor (AHR)^24,25,72,73^. AHR is a cytoplasmic bHLH-PAS transcription factor that can be activated by diverse chemicals. Upon ligand binding, and similarly to HIF-1α, AHR is translocated to the nucleus where it dimerizes with HIF-β (ARNT) and forms an active transcription factor that regulates the expression of several genes involved in detoxification, angiogenesis, cell proliferation, adhesion and migration, among others processes^74^. CYP1A1 is classically induced in response to AHR activation and, according to our results, the treatment of hypoxic MCF-7 cells with high concentrations of HT dramatically up-regulates the transcription of this protein involved in the metabolism of exogenous chemicals. These data appear to indicate that, at those concentrations, HT acts as an AHR ligand and promotes the expression of AM and VEGF through binding to their AHR-responsive elements. Although a complete view of the role of AHR in breast tumor growth is not currently available, AHR activation has been extensively linked with malignant transformation. In fact, a recent study in a cohort of 439 breast tumors showed that high AHR expression correlated with the up-regulation of genes involved in inflammation, metabolism, invasion and growth factor signaling while high AHRR mRNA levels correlated with good-metastasis free survival^26^. Ligands for AHR are diverse and include not only pharmaceuticals but also dietary compounds such as phenols, natural and synthetic flavonoids^75^. The rank of concentrations at which those compounds exert such effect is highly variable and depends on the chemical structure but also on the biological model used^76^. Our structural modeling of HT binding to AHR supports that this phenol can act as an AHR ligand. In fact, the binding poses found show a π-π interaction proved to be crucial for TCDD binding, together with interactions with residues already shown to be important for TCDD binding to AHR^36,39^. Moreover, we also addressed the importance of concentration in the AHR agonist activity of HT, as only high levels of this phenol, clearly over its dietary intake, are able to induce CYP1A1. A deeper *in silico* analysis, going from less computationally intensive methods, like MMGBSA^77^, to other requesting much more computational resources such as alchemical free energy calculations^78,79^, should be necessary to shed light into the molecular basis of the different expected binding free energy of HT as compared to TCDD. However, the fact that the induction of AM and VEGF by HT is not completely abolished in ARNT-silenced cells suggests the involvement of other mechanisms additional to HIF-1 and AHR. ARNT2 is homologous to ARNT protein. Its expression is restricted to neural tissues and the kidney, but it is also found in multiple cancer cells. Although both ARNT and ARNT2 bind equally with HIF-α subunits, ARNT is much more efficient that ARNT2 in the AHR-mediated up-regulation of CYP1A1^80^. Therefore, although further experiments should be carried out, it could be hypothesized that the transcriptional effect of HT may be exerted through a combination of the AHR-ARNT and HIF-1α-ARNT/ARNT2 pathways.

To our knowledge, few papers compare the *in vitro* and *in vivo* antitumoral effect of HT in breast cancer. According to the literature, although very high concentrations of HT are necessary to inhibit the proliferation of breast cancer cells *in vitro*, a 0.5 mg HT/kg/day dose is able to significantly reduce breast tumor volume in rats^81^. In one study with breast cancer patients, 15 mg HT/day exerted a positive effect in women that received different cycles of combined chemotherapy of epirubicin and cyclophosphamide^82^. HT absorption is good but its total bioavailability is only 5–10%. Therefore, although 100 or 200 µM are unachievable concentrations with diet intake or nutraceutical presentations of HT, the *in vivo* results seem to indicate that lower doses can attain similar results to those obtained *in vitro* with higher concentrations. The hypoxic environment is crucial in a tumoral context but its particular impact on in the response to anticancer treatments has been hardly analyzed. Our study aims to be a first step to shed light into the impact of hypoxia on the molecular response mediated by this phenol. The data we present appear to indicate that, in a hypoxic context, HT modulates different molecular mechanisms involved in tumor malignancy and resistance to therapy. The inhibition of the PI3K/Akt/mTOR pathway can overcome resistance to hormonal and anti-HER2 targeted therapies^83^, and HIF inhibitors are also promising molecules in cancer treatment^84^. Therefore, our data support that HT could have a chemomodulatory effect in breast cancer. It has already been shown, in an *in vivo* rat breast cancer model, that the co-treatment with paclitaxel and HT (0.5 mg/kg/day) decreases systemic oxidative stress, tumor volume and cell proliferation^81^. It would be plausible to hypothesize that some of the molecular mechanisms proposed here could underlie such response.

In conclusion, our results suggest that HT decreases the PI3K/Akt/mTOR pathway and HIF-1α in hypoxic MCF-7 cells. Moreover, we describe for the first time that high doses of HT, recurrently used in the literature, may act as an AHR agonist favoring the induction of angiogenic genes under hypoxic conditions. Therefore, our results provide new insights into the effect of HT in a hypoxic environment, and point to the importance of concentration in the comprehensive analysis of the biological potential of this compound.

## Methods

### Chemicals and reagents

HT (purity ≥98%) was obtained from Extrasynthese, France. Dulbecco’s modified Eagle’s medium (DMEM) and sodium pyruvate were from Capricorn Scientific, Germany, foetal bovine serum (FBS) was from Sigma, USA, and Trypan Blue Solution, 0.4% from Thermo Fisher, USA. Primary antibodies mTOR (2972 S), p-mTOR (2971 S), S6 (2217 S) and p-S6 (2211 S) were purchased from Cell signaling Technology, USA; HIF-1α (A300–286A) from Bethyl, USA; PARP-1 (C-2-10) from Calbiochem, Germany; anti-poly(ADP-ribose) (α-PAR) (4355-MC) from Trevigen, USA; α-Tubulin antibody (T5168) from Sigma, USA and FIH-1 (sc-26219) from Santa Cruz, USA. Apoptosis was quantified using the FITC Annexin V Apoptosis Detection Kit with PI (ANXVKF, Immunostep, Spain). RNA was isolated using the RNeasyPlus Mini kit (Qiagen, Germany). cDNA Synthesis Kit for RT-qPCR and iTaq UniverSYBR for Real-time PCR were from Bio-Rad, USA. Primers were synthesized by Biomedal S.L. (Spain). ARNT siRNA (s1613 and s1615), scramble siRNA (sc-37007) and the transfection reagent jetPRIME were from Ambion, Santa Cruz and Polyplus Transfection, USA, respectively. HIF-1α siRNA was from Sigma (forward 5′-CUGAUGACCAGCAACUUGA-3′, reverse 5′-UCAAGUUGCUGGUCAUCAG-3′).

### Cell culture and treatments

Human breast cancer MCF-7 cells were grown in 10% foetal bovine serum and 1% sodium pyruvate supplemented DMEM at 37 °C in 5% CO_2_ and 21% O_2_. Cells were pre-treated or not with different concentrations of HT, prepared in ethanol immediately before use, for 16 h under normoxic conditions (21% O_2_), being cultured during the last 4 h either in normoxic or hypoxic conditions (1% O_2_). Control cells were treated with an equal ethanol concentration.

### Cell viability assay

The ratio of viable cells in each experimental condition was quantified by the trypan blue exclusion assay in a TC20™ Automated Cell Counter (BioRad, USA) according to manufacturer´s recommendations. Cell viability was expressed as ratio of viable (non-stained) cells relative to normoxic non HT-treated cells.

### Annexin V- propidium iodide double-staining assay

The induction of apoptosis in MCF-7 cells was evaluated by using a FITC Annexin V Apoptosis Detection Kit with propidium iodide (PI) (ANXVKF, Immunostep, Spain) according to the manufacturer’s protocol. Data acquisition and analysis were carried out using a flow cytometer (LSR Fortessa, BD Bioscience) and the BD FACSDiva^TM^ software. Cells in upper left column (Q1) represent necrotic cells (Annexin V negative and PI positive cells); cells in upper right column (Q2) represent late apoptotic cells (Annexin V and PI positive cells); cells in lower left quadrant (Q3) represent viable cells (Annexin V and PI negative cells); cells in lower right quadrant (Q4) represent early apoptotic cells (Annexin V positive and PI negative cells).

### Measurement of nitric oxide level

Nitric oxide (NO) level was indirectly quantified by determining nitrate/nitrite and S-nitroso compounds (NOx), using an ozone chemiluminescence-based method. For this purpose, cells of each experimental condition were collected and lysed by 3 freeze–thaw cycles. After centrifugation at 14000 g for 30 min, the supernatants were collected and protein was quantified. Samples were deproteinized in a deproteinization solution (0.8 N NaOH and 16% ZnSO_4_). The total amount of NOx in the deproteinized samples was determined the purge system of Sievers Instruments, model NOA 280i. NOx concentrations were calculated by comparison with standard solutions of sodium nitrate. Final NOx values were referred to the total protein concentration in the initial extracts.

### Western blot

For western blot analysis, equal amounts of denatured total-protein extracts (20 μg) were loaded and separated on a 7.5% (HIF-1α, p-mTOR, α-PAR and PARP-1), or 10% (FIH-1 and p-S6) SDS-polyacrylamide gel. Proteins in the gel were transferred to a PVDF membrane (Amersham Pharmacia Biotech) and then blocked. Monoclonal antibodies to HIF-1α (1/5000), m-TOR (1/500), p-mTOR (1/1000), PARP-1 (1/1000), α-PAR (1/5000), FIH-1 (1/7000), S6 (1/1000), p-S6 (1/5000) and to α-tubulin (1/20.000), as a loading control, were used for detection of the respective proteins. Antibody reaction was revealed by means of chemiluminescence detection procedures according to the manufacturer’s recommendations (ECL kit, Amersham Corp). Western-blot was quantified by using TotalLab software.

### Quantitative Real-time PCR (qRT-PCR)

Real-time PCR was performed in a CFX384 Touch Real-Time PCR Detection System (Bio-Rad) using iTaq UniverSYBR. The sequences of the primers are below: *HIF-1α*, forward 5′-TGCTTTAACTTTGCTGGCCC-3′, reverse 5′-GTTTCTGTGTCGTTGCTGCC-3′; *AM*, forward 5′-CTCTGAGTCGTGGGAAGAGG-3′, reverse 5′-CCCTGGAAGTTGTTCATGCT-3′; *VEGF*, forward 5′-TTGTACAAGATCCGCAGACG-3′, reverse 5′-TCACATCTGCAAGTACGTTCG-3′; *GLUT-1*, forward 5′-AGGCTTCTCCAACTGGACCT-3′, reverse 5′-CCTCGGGTGTCTTGTCACTT-3′; *LDHA*, forward 5′-AGGCTACACATCCTGGGCTA-3′, reverse 5′-CCCAAAATGCAAGGAACACT-3′; *Oct-4*, forward 5′-GGCTCGAGAAGGATGTGGTC-3′, reverse 5′- CCAGCAGACACCTTAGACGA-3′; *AHRR*, forward 5′-GAAGGAGCAGCAGAGAGAGC-3′, reverse 5′- CTTTGTGGGTCCTGGAGTCT-3′; *CYP1A1*, forward 5′-CAAGGGGCGTTGTGTCTTTG-3′, reverse 5′- GTCGATAGCACCATCAGGGG-3′; *PPIA*, forward 5′-TTCATCTGCACTGCCAAGAC-3′, reverse 5′- TCGAGTTGTCCACAGTCAGC-3′. Experiments were performed in triplicate, and the relative quantities of target genes, corrected with the normalizing gene PPIA, were calculated using the Bio-Rad CFX Manager Software.

### HIF-1α and ARNT siRNA transfection

HIF-1α, ARNT and Scr siRNAs were transfected with transfection reagent according to the manufacturer’s instructions. Briefly, MCF-7 cells (11 × 10^4^/well) were plated into 6-well plates, allowed to adhere for 24 h and incubated with the siRNAs for 24 h. Different concentrations of each siRNA were initially tested (20, 40 and 60 nM of siHIF-1α; 5, 10 and 25 nM of siARNTs). Finally, siHIF-1α 40 nM, siARNTs 25 nM and similar concentrations of Scr-siRNA were used. Two pairs of ARNT siRNAs were used to ensure a maximum silencing. The transfection medium was then replaced with fresh medium for 24 h before further treatment with HT and/or hypoxia. The efficiency of silencing was evaluated by western-blot analysis of HIF-1α and ARNT.

### Modelling of HT binding to AHR

The model of the human AHR PAS-B domain was obtained by homology modelling using as templates three holo structures of the HIF-2α PAS-B domain, with PDB IDs 3F1O^30^, 3H7W^31^ and 3H82^85^. The Prime module of the Schrödinger Maestro 2017-1 suite was used to obtain an homology model using the Consensus Homology Model, using as query sequence residues 284–390 of human AHR. The model was subsequently loaded into the web program ProteinPrepare^86^, which protonates at pH=7 and optimizes the hydrogen bond network. The model for HT was manually drawn and imported into UCSF Chimera^87^, where its structure was optimized for 100 steepest-descent followed by 100 conjugate-gradient steps. The HR model, together with the human AHR one, were on the one hand uploaded into Bindscope^34^, and on the other one prepared with the DockPrep utility of Chimera to be used in a docking calculation with Autodock Vina^33^. Amber ff14SB^88^ charges were used for the protein, whilst AM1-BCC^89,90^ charges were assigned to HT. The binding pocket used, which is the same found in other docking experiments, was determined with DeepSite^91^. The cartesian coordinates of the center of the binding pocket found with DeepSite were used to define a search box for Autodock Vina of 10×10×10 Å^3^, and for other parameters, defaults were used. Figures were rendered with UCSF Chimera and Maestro.

### Statistical analysis

Data are expressed as means ± SD of at least three independent experiments. Statistical comparisons between the different experimental groups and their corresponding controls were made with Student’s t-test, accepting p < 0.05 as the level of significance, using GraphPad Prism 6 software (GraphPad Software Inc.).

### Experimental methods guidelines statement

All experiments were performed in accordance with relevant guidelines and regulations.

## Supplementary information

Supplementary Fig. 1.

Dataset 1.

## Data Availability

The datasets generated during and/or analysed during the current study are available from the corresponding author on reasonable request.
