# Supplementary material for: Crosstalk between hydroxytyrosol, a major olive oil phenol, and HIF-1 in MCF-7 breast cancer cells
Source: Sci Rep. 2020 Apr 14;10:6361. doi: 10.1038/s41598-020-63417-6 (PMC7156391; doi:10.1038/s41598-020-63417-6)
Supplement: Supplementary file 1 — Supplementary Fig. 1. [file 41598_2020_63417_MOESM1_ESM.pdf]

Crosstalk between hydroxytyrosol, a major olive oil phenol, and HIF-1 in MCF-7 breast cancer cells

Jesús Calahorra[1], Esther Martínez-Lara[1], José M. Granadino-Roldán[2], Juan M. Martí[3], Ana Cañuelo[1], Santos Blanco[1], F. Javier Oliver[3], and Eva Siles*[1]

[1] Departamento de Biología Experimental Universidad de Jaén, Campus Las Lagunillas s/n, Jaén, 23071, Spain
[2] Departamento de Química Física y Analítica, Universidad de Jaén, Campus Las Lagunillas s/n, Jaén, 23071, Spain.
[3] Instituto López Neyra de Parasitología y Biomedicina, IPBLN, CSIC PTS-Granada, Armilla, 18016, Spain.
*esiles@ujaen.es

Supplementary Information

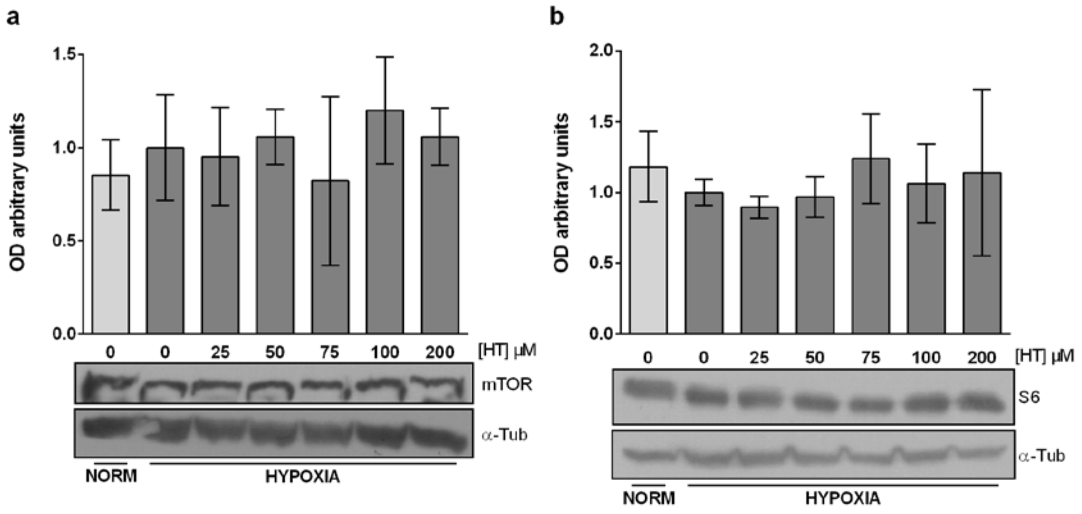

Supplementary Figure 1. Densitometric quantifications of mTOR (a) and S6 (b) relative to α-tubulin protein level (α-Tub). Values represent the mean ± SD from three independent experiments.
